# Catabolic protein degradation in marine sediments confined to distinct archaea

**DOI:** 10.1038/s41396-022-01210-1

**Published:** 2022-02-26

**Authors:** Xiuran Yin, Guowei Zhou, Mingwei Cai, Qing-Zeng Zhu, Tim Richter-Heitmann, David A. Aromokeye, Yang Liu, Rolf Nimzyk, Qingfei Zheng, Xiaoyu Tang, Marcus Elvert, Meng Li, Michael W. Friedrich

**Affiliations:** 1 Microbial Ecophysiology Group, Faculty of Biology/Chemistry, University of Bremen, Bremen, Germany; 2 MARUM - Center for Marine Environmental Sciences, University of Bremen, Bremen, Germany; 3 Max Planck Institute for Marine Microbiology, Bremen, Germany; 4 School of Resources and Environmental Engineering, Anhui University, Hefei, Anhui China; 5 Institute of Chemical Biology, Shenzhen Bay Laboratory, Shenzhen, China; 6 Archaeal Biology Center, Institute for Advanced Study, Shenzhen University, Shenzhen, China; 7 Shenzhen Key Laboratory of Marine Microbiome Engineering, Institute for Advanced Study, Shenzhen University, Shenzhen, China; 8 School of Chemical Biology and Biotechnology, Peking University Shenzhen Graduate School, Shenzhen, China; 9 Faculty of Geosciences, University of Bremen, Bremen, Germany

**Keywords:** Biogeochemistry, Microbial ecology

## Abstract

Metagenomic analysis has facilitated prediction of a variety of carbon utilization potentials by uncultivated archaea including degradation of protein, which is a wide-spread carbon polymer in marine sediments. However, the activity of detrital catabolic protein degradation is mostly unknown for the vast majority of archaea. Here, we show actively executed protein catabolism in three archaeal phyla (uncultivated Thermoplasmata, SG8-5; Bathyarchaeota subgroup 15; Lokiarchaeota subgroup 2c) by RNA- and lipid-stable isotope probing in incubations with different marine sediments. However, highly abundant potential protein degraders Thermoprofundales (MBG-D) and Lokiarchaeota subgroup 3 were not incorporating ^13^C-label from protein during incubations. Nonetheless, we found that the pathway for protein utilization was present in metagenome associated genomes (MAGs) of active and inactive archaea. This finding was supported by screening extracellular peptidases in 180 archaeal MAGs, which appeared to be widespread but not correlated to organisms actively executing this process in our incubations. Thus, our results have important implications: (i) multiple low-abundant archaeal groups are actually catabolic protein degraders; (ii) the functional role of widespread extracellular peptidases is not an optimal tool to identify protein catabolism, and (iii) catabolic degradation of sedimentary protein is not a common feature of the abundant archaeal community in temperate and permanently cold marine sediments.

## Introduction

Metagenomic approaches have substantially expanded the known microbial diversity and revised our understanding of evolution of life [1–3]. The phylogeny of archaea in the tree of life provides strong links to understand the prokaryote-to-eukaryote transition [4–6], but their metabolic capabilities and ecological roles are rarely reported. Within a decade, novel archaeal phyla such as Thermoplasmatota [7], Bathyarchaeota [8] and Asgard archaea [6, 9] have been discovered in various environments. These phyla are highly abundant in sediments, but many of their affiliated subgroups are still microbial “dark matter” with respect to the unknown physiological activities, owed to difficulties to cultivate them under laboratory conditions. Only few studies have reported carbon and energy utilization modes for some of these recently discovered archaea [4, 10–12], but up to date, metagenome analysis is still the predominant way to predict their physiological capabilities. For example, Bathyarchaeota and some Thermoplasmata possess genes encoding fatty acid oxidation and protein degradation [13–17], and many subgroups of Asgard archaea and Bathyarchaeota may be able to utilize a variety of organic carbon sources [6, 18–20]. However, “protein degradation” is a rather undefined term as it leaves open whether microorganisms make a living of the protein for energy generation (catabolism; amino acid degradation) or for anabolism (amino acid assimilation).

Considering the presence of extracellular DNA in sediments and substrate-dependent regulation of gene expression in cells [21], metagenomic analysis reflects potentials, yet precludes inferring microbial activity in the environment. This is especially challenging when different substrate utilization modes occur concurrently. One such example is mixotrophic organic and inorganic carbon utilization (demonstrated for some Lokiarchaeota and Bathyarchaeota [10–12]), another is the cellular lipid metabolism, which depends on environmental conditions such as temperature and pH [22], and for which *de novo* synthesis and scavenging from sediments is possible and potentially carried out simultaneously [23]. In this respect, metagenomic analyses are limited in predicting the active use of encoded metabolic pathways by microorganisms.

Such inferences from metagenome-assembled genomes (MAGs) have predicted that some archaeal groups such as Asgard archaea and Bathyarchaeota are potential protein degraders due to the presence of genes encoding extracellular peptidases [13, 18]. However, it is still unknown if these archaea i) are actively involved in extracellular protein utilization and ii) if proteins are utilized as both energy and carbon sources, i.e., in catabolism and anabolism of amino acids. For example, Thaumarchaeota are predicted to degrade detrital protein [24], but these archaea seem to rely on acquisition of ammonia from amino acids for energy metabolism and assimilate carbon into biomass only as carbon source [25–28]. Considering these findings, we hypothesized that the utilization of proteins as both, carbon and energy source, thus in catabolic and anabolic fashion, is not a common feature for all archaea, which are equipped to assimilate protein into their biomass, including nucleic acids and lipids. In order to address this hypothesis, we applied RNA based stable isotope probing (SIP) with its ultra-high sensitivity for identifying the activity of uncultivated microbes [29]. A combination of ^13^C-labeled and unlabeled substrates, i.e., protein and dissolved inorganic carbon (DIC), was used for RNA-SIP in order to probe the potential for mixotrophy, i.e. thriving on both, organic carbon substrates and DIC, a life strategy which is an increasingly recognized for archaea [10, 30]. Selective amendment of antibiotics to suppress possibly competing bacteria, as well as analysis of archaeal MAGs allowed the systematic detection and analysis of active catabolic archaeal protein degraders [25]. Such incubations also allowed us to probe the activity of lipid biosynthesis using lipid-stable isotope probing (lipid-SIP) to unveil the transformation of the protein-derived carbon to lipids.

## Materials and methods

### Sediment incubation for SIP

For incubations, temperate (Helgoland mud area) and permanently cold (Cumberland Bay) marine sediments were used. Sediment from Helgoland mud area (54°05.23’N, 007°58.04’E; water depth: 27.9 m) was collected by gravity cores during the RV HEINCKE cruise HE443 in 2017. Based on geochemical profiles [31], sediment from sulfate reduction zone and methanic zone i.e., 16–41 cm and 238–263 cm below subsurface was used for incubations (Table S1). Similarly, permanently cold marine sediment sediments from Cumberland Bay were retrieved with gravity cores (54°15.899’S, 36°26.248’W; water depth: 253 m) during the RV METEOR expedition M134 to South Georgia Island in 2017. Sediment cores for all sites were sectioned into 25-cm sections, which were homogenized and stored anoxically in 2.6 L jars at 4 °C until use. Geochemical profiles and sampling were previously described elsewhere [32]. In order to prepare incubations for protein degradation, ^13^C-labeled protein was obtained by growing *E.coli* DSM 498 strain in ^13^C-labeled *E.coli*-OD2 C medium (^13^C, 98%, Silantes, Germany) [33]. Protein was extracted as described previously [12]. DNA contamination (<0.6 ng/µl in all cases) was checked with the Quant-iT PicoGreen assay (Invitrogen, Eugene, Oregon, USA). Sediment and artificial seawater (w:v = 1:4, 50 ml) were homogenized, incubated anaerobically in 120-ml serum flasks, followed by a 10-day pre-incubation considering a different incubation temperature (10 °C) compared to the storage temperature (4 °C). Triplicate setups with a combination of ^13^C-labeled and unlabeled carbon sources (protein and DIC) were performed (Table S1), for which 10 mM bicarbonate and 100 µg protein were amended according to the background concentrations of protein and DIC in sediments [34, 35]. Unlabeled and ^13^C-labeled DIC controls of slurry incubations without protein were also prepared in parallel. The treatments amended with antibiotics were also carried out using only streptomycin (100 mg/l) and a combination of five antibiotics (streptomycin, ampicillin, kanamycin, vancomycin and D-cycloserine: 50 mg/l each – see Table S1). The development of δ^13^C-CO_2_ in headspace was tracked as indicator for stopping incubations after 24 to 51 days (see Table S1 for details of incubation time).

### Nucleic acid SIP

Nucleic acids were extracted according to Lueders et al. [36]. Briefly, sediment samples were treated by bead beating, followed by purification using phenol-chloroform-isoamyl alcohol (25:24:1) and chloroform-isoamyl alcohol (25:1). Nucleic acids were precipitated with polyethylene glycol 6000 (~30%). For RNA extraction, DNA was removed according to the RQ1 DNase kit (Promega, Madison, Wisconsin, USA) at 37 °C for 30 min. After digestion by DNase, RNA was purified by using phenol-chloroform-isoamyl alcohol and chloroform-isoamyl alcohol, as well as precipitated by polyethylene glycol again. A final volume of 100 µl RNA samples was retrieved. RNA was quantified fluorometrically based on Quant-iT RiboGreen (Invitrogen, Eugene, Oregon, USA). Triplicate RNA extracts were combined in order to obtain sufficient amounts of RNA for SIP. Isopycnic centrifugation and gradient fractionation were performed according to the methods previously described [36]. In detail, about 0.5–1 µg RNA were added to gradient medium containing 6 ml CsTFA (GE Healthcare, Buckinghamshire, UK) and 240 µl formamide. After ultracentrifugation at 124,000 *g* for 65 h, 12-13 fractions (~410 µl) were obtained from each sample. Reverse transcription of RNA to cDNA was conducted using GoScript reverse transcription kit (Promega, Madison, Wisconsin, USA). cDNA from fractions 4 and 5 (heavy), 6 and 7 (middle), 8 and 9 (light), as well as 10 and 11 (ultra-light) were combined for sequencing, including SIP fractions from the ^13^C-treatments and unlabeled controls. PCR employing KAPA HiFi HotStart PCR kit (KAPA Biosystems, Cape Town, South Africa) was performed with barcoded archaeal primer Arc519F (5’-CAGCMGCCGCGGTAA-3’) [37] and Arch806R (5’-GGACTACVSGGGTATCTAAT-3’) [38]. DNA amplification, PCR products purification and library preparation were described previously [39]. Amplicons were sequenced on NovaSeq 6000 platform (2 × 250 bp, Illumina) at Novogene (Cambridge, UK). Raw reads were processed using the QIIME 1.9.0 software package according to a previous study with modifications [39]. In brief, joined forward and reverse reads were quality filtered to a minimum length of 242 bp, followed by de-replication, removal of singletons and chimeric sequences. Sequence OTUs were clustered at 97% identity using UPARSE-OTU [40]. Taxonomy was assigned based on the SILVA 132 database [41].

SIP criteria were applied to define ^13^C-labeling of RNA in heavy fractions according to a previous study [12]. Inter-gradient subtraction values were calculated using the relative abundances of sequences in the heavy and light fractions from ^13^C-labeled (^13^C_Heavy_, ^13^C_Light_) and unlabeled (^12^C_Heavy_, ^12^C_Light_) treatment: (^13^C_Heavy_ – ^13^C_Light_) – (^12^C_Heavy_ – ^12^C_Light_), for which both ^13^C-labeled and unlabeled controls were considered. Due to the low background of Thermoplasmata and Loki-2c, an increase of 0.5% in inter-gradient subtraction value was regarded as ^13^C-labeling of RNA by ^13^C-substrate incorporation. For Bathy-15 with a high background in the original sediment samples, a more than 5% inter-gradient subtraction value indicated ^13^C-labeling [12] (Fig. S1).

### Lipid-SIP

Lipid-SIP is highly sensitive to quantify low amounts of assimilated carbon, thereby facilitating the identification of microorganisms and the detection of lipid biosynthetic pathways [42, 43]. Total lipids were extracted from the freeze-dried sediments of SIP samples (~3 g) using a modified Bligh-Dyer protocol [44]. In brief, a mixture of methanol, dichloromethane and twice phosphate and twice trichloroacetic acid buffer were used for extraction by sonication for 10 min. The combined lipid extracts were washed 3 times with water to remove the remaining buffer. Finally, the total lipid extract (TLE) was evaporated under a stream of nitrogen. The isoprenoidal derivatives of diether and tetraether lipids (i.e. phytane and biphytanes) were obtained from the TLE using ether-cleavage [45]. In brief, 300 µl BBr_3_ was added to the TLE under an argon atmosphere in glass vials, which were sealed and heated to 60 °C for 2 h. After reaction, 1 ml lithium triethylborohydride in tetrahydrofuran (1.0 M; Sigma Aldrich) was added in order to reduce bromides to hydrocarbons. Phytane and biphytanes were quantified by gas chromatrogaphy - flame ionization detection (GC-FID; Thermo Finnigan, Bremen Germany), followed by ^13^C composition measurements using a GC-isotope ratio mass spectrometer (IRMS) consisting of a Thermo Scientific Trace GC equipped with a Restek Rxi-5 ms column (30 m × 250 µm × 0.25 µm; Restek, Bad Homburg, Germany) and coupled via a GC Isolink interface to a DELTA V Plus IRMS system (Thermo Scientific, Bremen Germany). Temperature settings were as follows: initial oven temperature at 60 °C for 1 min, increase to 150 °C at a rate of 10 °C/min, increase to 310 °C at a rate of 4 °C/min, hold at 310 °C for 40 min; injector temperature 290 °C; oxidation reactor of the combustion interface 1000 °C. Isotopic values are reported in the delta notation as δ^13^C (‰) relative to the Vienna PeeDee Belemnite (VPDB) standard. The 1σ precision of repeated isotopic analysis (*n* = 2) based on the internal standard (tetracontane) was less than 1‰.

### Analysis of ^13^C-CO_2_

The δ^13^C values of CO_2_ in the headspace from the triplicated treatments were determined by injecting 1 mL gas sample into a Thermo Finnigan Trace GC connected via a GC III interface to a DELTA Plus IRMS (Finnigan MAT, Bremen, Germany) using chromatographic and temperature settings described previously [46]. Isotopic values are reported in the delta notation as δ^13^C (‰) relative to VPDB. The 1σ precision of repeated isotopic analysis (*n* = 3) of the standard CO_2_ gas was less than 1‰. Deviations of δ^13^C values were between 1 and ±100‰ (for DIC with ^13^C label uptake of >1500‰).

### Analysis of extracellular peptidase in archaeal MAGs

A total of 180 representative archaeal MAGs were used for the analysis of extracellular peptidase. In brief, a maximum of 5 representative archaeal MAGs for each lineage were retrieved from NCBI Genome and Assembly databases using ‘wget’ (July 2020) based on the archaeal classification a previous report [47]. For Thermoplasmata, MAGs from different order levels were used for analysis according to the previous study [48]. To search for peptidase, protein sequences for archaea were blasted against the MEROPS peptidase database with an e-value cutoff of 1E-20 as described elsewhere [49], and the extracellular peptidases were further determined by using SignalP software (5.0b), which has a good coverage for archaeal signal peptides [50]. Bray-Curtis dissimilarity for extracellular peptidases was calculated in R software (3.6.3) using the package ‘picante’.

### Metagenomic assembly, genome binning and gene annotation

A total of ~1 µg DNA extracted from the original samples collected from Helgoland Mud area sediments with different depths (16–41 cm, 50–75 cm and 238–263 cm) and Cumberland Bay sediment (225 cm) were used for metagenomic sequencing on the HiSeq 4000 platform (2 × 150 bp, Illumina) at Novogene (Cambridge, UK), generating at least 336.84 million clean reads and 2.1 million contigs. The previous SIP samples [12] were used for metagenomic analysis in which SG8-5 and Bathy-15 were identified (Fig. S2 and Fig. S3). For these SIP samples, a minimum of 151.5 million clean reads and 3.7 million contigs were retrieved, respectively. The metaWRAP package (1.2.1) [51] was employed to analyze the raw metagenomic reads. Briefly, quality checked reads were trimmed and then assembled using MEGAHIT(1.1.3) with the default settings [52]. Scaffolds above 1,000 bps were binned into refined genomic bins using a combination of MaxBin2 (2.2.6) [53], CONCOCT (1.0.0) [54] and metaBAT2 (2.12.1) [55]. To improve the quality of the bins, archaeal MAGs were remapped with the short-read mapper BWA (0.7.17) [56] and re-assembled using SPAdes (3.13.0) [57]. The completeness and contamination of MAGs were estimated by CheckM (1 .0.12) [58]. At least two MAGs with the best quality from each archaeal subgroups were analyzed, for most of them had a high completeness (>80%) and a contamination ratio below 6.4% (See Table S2 for detail MAG information). For Bathy-15 archaea, five MAGs were analyzed (50 to 75% completeness) (Table S2). Taxonomic classifications of archaeal MAGs were based on GTDB database [59]. Protein-coding regions were predicted using Prodigal (version 2.6.3) with the “-p meta” option [60]. The KEGG server (BlastKOALA) [61], eggNOG-mapper (5.0.0) [62], InterProScan tool (5.44–79.0) [63], and Diamond (0.9.22) vs. NCBI-nr database searched in April 2020 (E-value cutoff ≤1e-5) were used to annotate the protein-coding regions.

### Phylogenetic analyses

For a detailed phylogenetic analysis, a collection of archaeal 16S rRNA gene sequences was aligned using SINA Aligner [64]. These 16S rRNA gene sequences were retrieved from 16S rRNA gene OTUs from high throughput sequencing, clone sequences, 16S rRNA genes extracted from archaeal MAGs and archaea representative sequences obtained from ARB (Silva 138 database) [65]. Ribosomal RNA genes in the MAGs were extracted by Barrnap (version 0.3, http://www.vicbioinformatics.com/software.barrnap.shtml). Maximum-likelihood tree was inferred with RAxML (8.2.11) with rapid bootstrapping using the GTRGAMMA model [66]. The tree files were edited through iTOL software [67]. Calculation of identity of 16S rRNA gene clones (position of *E. coli* 109–806) was performed in ARB [65].

Classification of Bathyarchaeota subgroup was carried out by constructing RAxML tree using 16S rRNA gene sequences obtained from a previous study [68]. Maximum-likelihood tree was calculated and edited as described above.

The concatenated set of 36 ribosomal protein genes based on the hidden Markov model profile from Lee [69] were used for phylogenetic analyses in Anvi’o (6.1) [70]. Maximum-likelihood trees were built using IQ-TREE (1.6.12) [71] with the best-fit model and 1000 times ultrafast bootstrapping.

## Results

### Protein catabolism and transformation into RNA and lipids by distinct uncultivated archaea

**Fig. 1:**
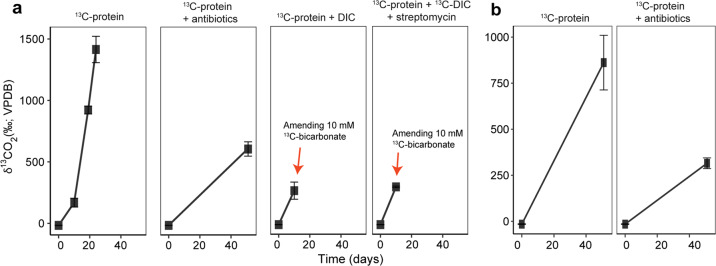
δ^13^C-CO_2_ in headspace of SIP incubations. The development of the stable carbon isotopic composition of δ^13^C-CO_2_ in incubations amended with ^13^C-substrates using (**a**) Helgoland mud and (**b**) Cumberland Bay sediment (*n* = 3, error bar = SD). Antibiotics indicate the mixture of streptomycin, ampicillin, kanamycin, vancomycin and D-cycloserine with 50 mg/l each.

**Fig. 2:**
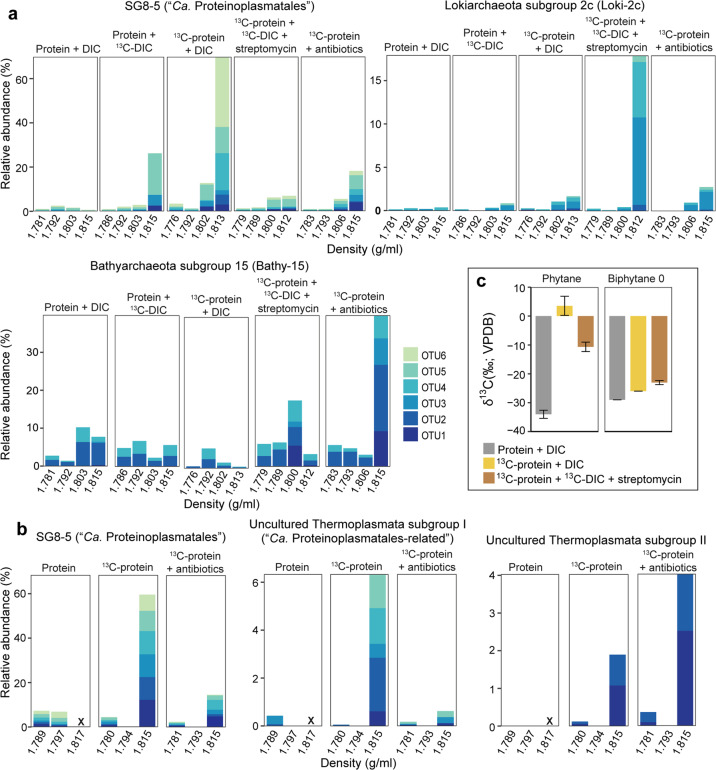
RNA-SIP and lipid-SIP targeting catabolic archaeal protein degraders. Relative abundance of 16S rRNA gene sequences of active archaeal protein degraders in total archaea from RNA-SIP gradient fractions in the Helgoland mud (**a**) and Cumberland Bay (**b**) sediment incubations. Active archaeal groups were identified based on the subtraction criteria (see Methods; Fig. S1) (**c**) δ^13^C values of phytane and biphytane 0 in sediment incubations (*n* = 2, error bar = SD). Phytane originates from archaeal diether lipids, mostly archaeol, and biphytane 0 from tetraether lipids, dominantly caldarchaeol.

In samples from the marine sediment of the Helgoland mud area, a range of archaeal groups such as Lokiarchaeota, Bathyarchaeota and Thermoplasmata were identified (Fig. S4a). To identify active protein-degrading archaea, we applied RNA-SIP using combination of ^13^C-labeled and unlabeled protein/bicarbonate, and antibiotics to suppress the canonical dominance of bacteria in enrichments [4, 25] (Table S1). The increasing δ^13^C-CO_2_ in the headspace of incubations indicated the breakdown of ^13^C-protein (Fig. 1). Such low δ^13^C-CO_2_ values (δ^13^C < 1500 ‰; ~2.7%) were insufficient to promote a density shift during RNA-SIP due to its high ^13^C threshold (10–20%) [72]. A fraction of five subgroups in total within three archaeal phyla, i.e., Thermoplasmatota (SG8-5 [73], Uncultured Thermoplasmata subgroup I and II), Lokiarchaeota (subgroup Loki-2c) and Bathyarchaeota (subgroup Bathy-15) were identified as active taxa that incorporated label in incubations with temperate and permanently cold marine sediment i.e., from Helgoland Mud Area (North Sea) (Fig. 2a, Fig. S5, Fig. S6) and Cumberland Bay (sub-Antarctic South Georgia island) (Fig. 2b). Lokiarchaeota and Bathyarchaeota were found active in Helgoland sediments only, whereas active Cumberland Bay communities were characterized by Thermoplasmata. Notably, SG8-5 was active in both sites, sharing a number of identical active OTUs, albeit under slightly different conditions: In Helgoland mud sediment incubations, we observed SG8-5 incorporating both inorganic carbon and protein as carbon sources into RNA (Fig. 2a). Amendment of antibiotics increased the enrichment of some archaea subgroups in the labeled RNA-fractions, indicating the suppression of bacteria activity (Fig. S4b). In these incubations, Loki-2c, a newly identified subgroup of Lokiarchaeota, and Bathy-15 (especially for OTU1) were identified as protein degraders in Helgoland mud sediment (Fig. 2a). These active OTUs including Thermoplasmata groups, Loki-2c and the OTU1 of Bathy-15 harbored a low abundance in unlabeled controls and original sediments (Fig. S4a). In contrast, OTUs affiliated to Loki-3 and Thermoprofundales [16] (MBG-D archaea) did not become labeled from ^13^C-protein or ^13^C-DIC in incubations despite their high abundances in the original sediments (Fig. S7).

Parallel to the RNA-SIP experiments, archaeal lipid SIP was carried out in order to i) trace the route of ^13^C-labeled substrate to other cellular macromolecules, i.e., membrane lipids and ii) tentatively identify the lipid composition of uncultivated archaea which is largely unknown since metagenomic inferences cannot fully elucidate lipid biosynthesis pathways [74]. Those populations strongly incorporating labeled substrates into RNA will likely direct ^13^C also to lipid synthesis, allowing an indirect identification of lipid content of these uncultivated archaea. We checked the active lipid biosynthesis of archaea in the samples “^13^C-protein + DIC” and “^13^C-protein + DIC + streptomycin”, but analysis was limited to these two samples as available biomass in other samples was predominantly used for RNA extraction. For incubations amended with ^13^C-protein and unlabeled DIC, in which RNA-SIP showed a strong stimulation of “*Ca*. Proteinoplasmatales” (up to 70% in the heavy fractions, Fig. 2a), a substantial shift in δ^13^C values of phytane (∆δ^13^C = 37.6‰) relative to the unlabeled control incubations indicated archaeol as the main ether lipid produced (Figs. 2a and 2c). For incubations amended with ^13^C-protein, ^13^C-DIC and streptomycin in which we observed a dominance of Loki-2c (~17% in the heavy fractions) and Bathy-15 (~18% in the heavy fractions), and phytane δ^13^C values (∆δ^13^C = 23.4‰) are simultaneously increasing with biphytane without cyclopentane moieties (biphytane 0, ∆δ^13^C = 6‰). This suggests that these archaea are synthesizing both archaeol- and caldarchaeol-based lipids during protein degradation (Figs. 2a and 2c).

### Pathways for extracellular protein degradation were found in more archaea than the active ones

**Fig. 3:**
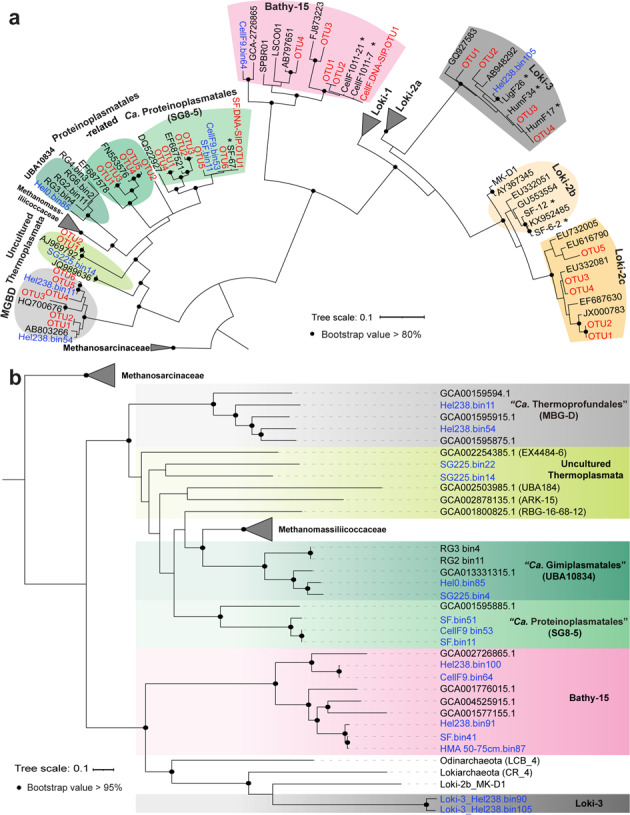
Maximum-likelihood phylogeny of uncultivated archaea. Maximum likelihood tree of (**a**) archaeal 16S rRNA genes and (**b**) of 36 concatenated ribosomal proteins. OTUs and MAGs obtained in this study are marked in red and blue, respectively. *Indicates clone sequences obtained from our previous study [12]. SF.DNA-SIP_OTU1: “*Ca*. Proteinoplasmatales” OTU identified in DNA-SIP samples in ^13^C-DIC/sulfur/lepidocrocite incubations; ^13^C-DIC.SIP.OTU1: Bathy-15 OTU identified from RNA-SIP samples in ^13^C-DIC/cellulose/lepidocrocite incubations (Fig. S2, S3; see supplemental Discussion 3).

We analyzed MAGs from original sediments and enrichment incubations for identifying the genetic equipment for protein degradation encoded in both labeled and unlabeled populations. For SG8-5, we did not retrieve MAGs from original samples but found SG8-5 MAGs with high quality from our previous DNA-SIP samples [12] (Table S2). We made sure that the analyzed MAGs were phylogenetically close to the identified catabolic ^13^C-protein degraders using single and multi-locus gene trees of the 16 S rRNA gene and ribosomal proteins respectively [69] (Fig. 3). We retrieved 12 archaeal MAGs including Uncultured Thermoplasmata, “*Ca*. Gimiplasmatales” [48] (UBA10834), SG8-5 and Bathy-15 (Fig. 3b, Table S2). According to the taxa descriptions for uncultured microorganisms [75], we propose “*Candidatus* Proteinoplasmatales” as the new name for the order of SG8-5 based on demonstrated active protein utilization by representatives of this archaeal subgroup, and the sister cluster of SG8-5 i.e., Uncultured Thermoplasmata subgroup I as “*Ca*. Proteinoplasmatales-related” (Fig. S8, Supplemental Discussion 1 and 2). In addition, OTUs that were initially classified as Odinarchaeota were re-assigned to the Lokiarchaeota as subgroup Loki-2c due to high similarities with 16S rRNA gene sequences (identity = ~92%) of Loki-2b (Fig. 3a, Fig. S9, Table S3 and Supplemental Discussion 1).

**Fig. 4:**
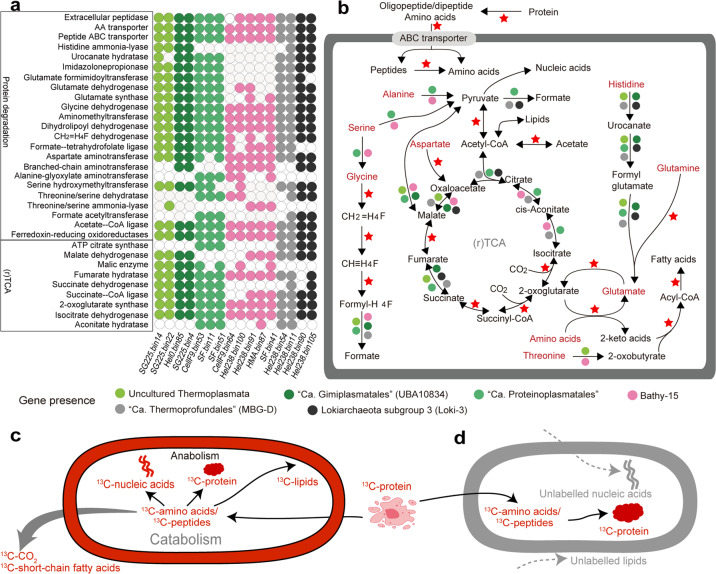
Pathways of extracellular protein degradation by uncultivated archaea. Gene presence (**a**) and pathways (**b**) involved in protein and amino acids in archaeal MAGs. Red star denotes gene presence in all analyzed MAGs. Conceptual framework for catabolic (**c**) and anabolic (**d**) protein utilization.

The pathways for protein and amino acid degradation encoded in archaeal MAGs were analyzed. For label incorporating populations, MAGs of Uncultured Thermoplasmata, “*Ca*. Gimiplasmatales” (UBA10834), *“Ca*. Proteinoplasmatales” and Bathy-15 and for populations not incorporating label from ^13^C-protein MAGs of MBG-D and Loki-3 archaea were used for annotation. Completeness of most MAGs was above 80% with a maximum contamination of 6.5% (Table S2). A range of functional genes involved in protein degradation was detected (Fig. 4a, Table S4 and Fig. S10) including extracellular peptidases, ABC transporters for peptide and amino acids, aminotransferases, 2-keto acids oxidoreductase and acetate-CoA ligase, associated with peptidase transport, degradation of individual amino acids and short-chain fatty acid formation (formate, acetate and others), respectively. Both labeled and unlabeled archaea encode a variety of pathways for catabolic amino acid degradation, including serine, aspartate, glutamate, glutamine, alanine and histidine and core genes for potential inorganic carbon assimilation (Fig. 4b, Fig. S11, Fig. S12; see Supplemental Discussion 2 for details of inorganic carbon incorporation).

**Fig. 5:**
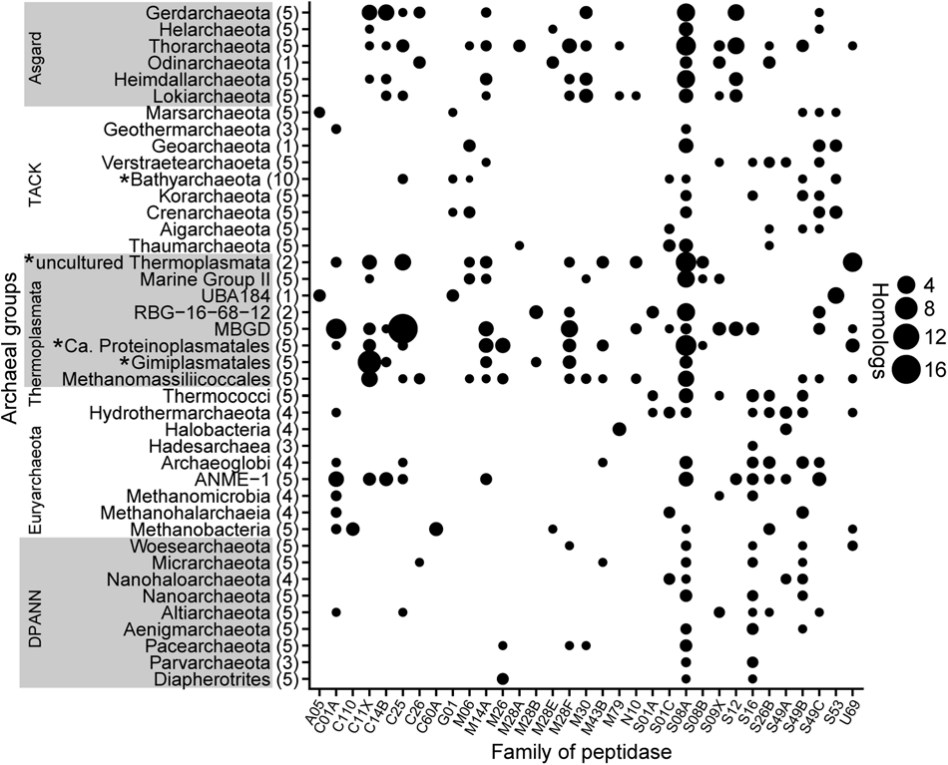
Distribution of the average amount of homologs encoding extracellular peptidases per MAG. Only peptidase families with at least one coding gene across all MAGs are shown. The number of archaeal MAGs used for analysis was provided after the name of each archaeal lineage. *Indicates contain of MAGs retrieved from this study.

MAGs representing non-labeled populations (MBG-D and Loki-3) featured a similar genetic blueprint for protein degradation, including a variety of extracellular peptidases (Fig. S7, Fig. S10). This finding triggered us to examine if extracellular peptidase genes are even more widespread among uncultivated archaea than were previously described [13] since archaeal diversity has been substantial expanded in recent years based on metagenomics. Therefore, we expanded our analysis to a large set of 180 archaeal MAGs retrieved from public databases (Table S5). A diverse set of extracellular peptidase genes, mainly spanning 32 peptidase families, were found broadly distributed in all analyzed archaea including DPANN, Euryarchaeota, Thermoplasmata, TACK and Asgard archaea, although SignalP annotation might underestimate the number of gene coding extracellular enzymes for archaea. Peptidase genes were more diverse and present in higher amounts of homologs in Thermoplasmata and Asgard archaea compared to TACK and DPANN archaea, with Euryarchaeota in between (Fig. 5, Fig. S13). However, the diversity and amount of these homologs were divergent among different lineages within the same phylum level. In addition, known non-protein-degrading archaea, such as the well-known anaerobic methanotrophs ANME-1 and methanogenic Methanomassiliicoccales possess a higher number of extracellular peptidases than TACK and other Euryarchaeota archaea (Fig. 5).

## Discussion

Understanding the controls of the degradation of organic matter in marine sediments is a longstanding research question [76], and microorganisms have been implicated as one of the most important factors [77, 78]. More recently, it has been suggested that uncultivated archaea play a role in the degradation of protein in marine sediments [20]. Our study found that ^13^C-labeled protein was actively transformed to archaeal nucleic acids and lipids in different marine sediment incubations by distinct archaea, including *Ca*. Proteinoplasmatales, Uncultured Thermoplasmata group II, “Proteinoplasmatales-related”, Bathyarchaeota subgroup 15 and Lokiarchaeota subgroup 2c. Such detrital protein transformation was involved in amino acid catabolism and anabolism into biomass (Figs. 4c, d), indicating that these archaea utilize protein as both energy and carbon sources. However, we found only a limited number of archaeal groups performing catabolic protein utilization, which contrasts the wide distribution of amino acid degradation pathways and genes encoding extracellular peptidases [13], moreover, partial pathways or single genes are insufficient to signify that the process is actively used. Notably, the restriction to a few groups carrying out catabolic protein utilization was found in both tested sediment types, i.e., temperate and permanently cold sediments from Helgoland Mud Area and Cumberland Bay. Both sediments shared one archaeal group (“*Ca*. Proteinoplasmatales”) and limited diversity of additional archaeal groups that catabolize protein. This indicated that most uncultivated archaea in temperate and permanently cold marine sediments in our study were not stimulated by amendments of protein.

Members of five archaeal groups were identified as protein degraders, and in fact catabolizing the added ^13^C protein. Catabolism of protein is supported by (i) the formation of ^13^CO_2_ in protein amended incubations (Fig. 1), and (ii) SIP (Fig. 2). RNA and lipids became labeled from added ^13^C-protein, indicating that the ^13^C-labeled carbon entered the central carbon metabolism of the catabolizing archaea. Typically, the breakdown of amino acids proceeds via pyruvate and acetyl-CoA, the central intermediates of metabolism and precursors of nucleotide and lipid biosynthesis (Fig. 4b).

In marine sediments, lipid biosynthesis can be achieved by recycling archaeal lipids from the external pools or  *de novo* synthesis using various carbon sources such as amino acids, methyl compounds and inorganic carbon [4, 23, 30, 79]. Our lipid-SIP data show that protein-derived carbon was transformed into archaeal lipids. Comparison of strongly labeled populations in RNA-SIP with patterns of labeled lipids in the same incubations indicate that *Ca*. Proteinoplasmatales is likely dominated by diether lipids (archaeol) while Loki-2c and Bathy-15 contain both diether and tetraether lipids (archaeol and caldarchaeol) (Fig. 2). Archaeal lipid biosynthesis based on amino acids as precursors can be divided into two steps: i) amino acid dissimilation into the key intermediate acetyl-CoA and ii) lipid synthesis using mevalonate pathways [80]. For amino acid breakdown, the TCA cycle will serve as the main pathway by converting the intermediate, i.e., glutamate into acetyl-CoA, which can be used for lipids and nucleic acid synthesis. In this case, ^13^C-labeled amino acids derived from ^13^C-labeled protein will be dominantly degraded catabolically, and thus, this ^13^C carbon will fill the pool of intermediates, e.g. acetyl-CoA. Hence, protein-derived carbon conversion into other biomass (lipids and nucleic acid) indicates both amino acid dissimilation and re-assimilation of the intermediates. This further supports that the identified archaea are both anabolic and catabolic protein utilizers.

The presence of genes encoding extracellular peptidases, and peptide transport proteins in MAGs is often the reason to predict archaeal protein degradation in association with the downstream pathways of amino acid utilization [13, 16]. However, many archaea feature pathways for peptide and amino acid utilization but protein degradation does not seem to be their main energy metabolism and carbon source for forming biomass. A striking example of such a contrast between prediction *in silico* and activity in situ is provided by our experiment. Loki-3 and MBG-D archaea, highly abundant in the original sediment, were not actively incorporating ^13^C-label from added protein into their RNA (Fig. S4a), although both have been proposed as potential protein “degraders” by metagenomics analysis before (Figs. 4a, b) [12, 13]. The lack of ^13^C-protein incorporation by these archaea might be explained by the assimilation of detrital protein as the amino acid source for intracellular protein biosynthesis, while using other endogenous carbon sources rather than catabolizing protein (Figs. 4c, d). Indeed, Loki-3 archaea might participate in lignin degradation in marine sediments [12]. Other examples are Methanomassiliicoccales and anaerobic methanotrophs ANME-1, which harbor ample extracellular peptidases (Fig. 5) as well as pathways for amino acid utilization [81, 82]. However, these methanogenic archaea and anaerobic methanotrophs rely on methanogenesis or methanotrophy, respectively, while using amino acids for protein biosynthesis [83], for cell wall rearrangement during cell growth [84, 85], or uptake of certain amino acids as osmolytes [86]. Another explanation for the inactive MBG-D archaea might be their extremely low growth rate [20]. In fact, our analysis shows that the distribution of extracellular peptidase genes is diverse among subgroups within the same phylum. For example, the actively protein degrading *Ca*. Proteinoplasmatales in our study have extracellular peptidase families M14A (carboxypeptidase), M26 (metallopeptidase), M28F (aminopeptidase), S8A (subtilisin) and U69 (self-cleaving autotransporter protein) dominating in their MAGs (Fig. S10), while the known protein degraders of Marine Group II [87] mainly harbor family S8A (Fig. 5). Indeed, gene copy numbers and diversity of extracellular peptidase are apparently not a good proxy for predicting protein degradation in marine sediments. What follows is that the mere presence of genes encoding protein utilization in MAGs and consequently even the detection of transcripts is insufficient to indicate actively occurring catabolic protein degradation. A more direct way such as the extremely sensitive RNA-SIP approach for detecting the active microbes without cell doubling [29] is critical to reveal the activity of catabolic protein degradation by archaea.

Besides various environmental factors, microorganisms have been implicated as important controls of organic matter degradation in marine sediment [77, 88], e.g., bacterial species participate in protein utilization [89]. Our study has revealed that degradation of protein in marine sediments could be mediated in principle by a large number of archaeal taxa indicated by the widespread distribution of extracellular peptidase genes. However, only a distinct selection of archaea became active in incubations in catabolic fashion, namely “*Ca*. Proteinoplasmatales”, Uncultured Thermoplasmata, “Proteinoplasmatales-related”, Bathy-15 and Loki-2c with a comparatively low abundance. This has far reaching implications for our understanding of carbon cycling in marine sediments: (i) degradative potentials inferred by metagenomics do not necessarily reflect that active carbon turnover occurs in situ, (ii) the presence of certain taxa in marine sediments cannot be referred to as proxies for ongoing carbon turnover, at least not in catabolic fashion, and (iii) assimilation of carbon consumes only a fraction of catabolic degradation (in anaerobes up to ~10% of a carbon substrate is assimilated, 90% or more depending on energy yield of the pathway [90, 91]). On the one hand, absence of catabolic utilization of amino acids (from protein added) in our study for certain archaeal taxa is corroborated by suggested low protein carbon assimilation rates in marine sediments due to the high energetic costs of translation, thus the synthesis of new protein [90]. On the other hand, our data contradict a scenario of low protein carbon conversion rates in marine sediments [92], as we find a number of archaea capable of degrading protein under anaerobic conditions. Certainly, our incubations cannot capture the low biomass conditions of deeper marine sediment layers, but more efforts are necessary to link rates of carbon turnover to active microbial metabolism in situ for understanding the role and identity of uncultivated active archaea in deep sea sediment carbon cycling. Overall, our findings reveal that some low-abundant archaeal groups are involved in the catabolic degradation of protein in temperate and permanently cold marine sediments, thus, likely reflecting a lower capability for carbon turnover than suggested by the omnipresence of degradative genes. In the view of global carbon cycling, further efforts are needed to understand patterns of protein utilization by archaea through analyzing samples from geographically diverse marine sediments.

## Supplementary information

Supplemental materials

Supplemental tables

## Data Availability

The archaeal MAGs data are available in NCBI database under the project PRJNA505997 (Biosample SAMN14451653 and SAMN14451654) and PRJNA678468 (Biosample SAMN16802728 to SAMN16802739, SAMN20193292 and SAMN20193293). Sequencing data of SIP samples have been submitted to Short Reads Archive with accession numbers from SRR8607872 to SRR8607991, SRR11429436 to SRR11429462 and SRR15174500 to SRR15174492. Clone sequences have been deposited at GenBank with accession numbers of MK551261-MK551285.
