# Supplementary material for: Catabolic protein degradation in marine sediments confined to distinct archaea
Source: ISME J. 2022 Feb 26;16(6):1617–26. doi: 10.1038/s41396-022-01210-1 (PMC9123169; doi:10.1038/s41396-022-01210-1)
Supplement: Supplementary file 1 — Supplemental materials [file 41396_2022_1210_MOESM1_ESM.docx]

**Catabolic protein degradation in marine sediments confined to distinct archaea**

Xiuran Yin^1,2,3†*^, Guowei Zhou^1,4†*^, Mingwei Cai^5,6†^, Qing-Zeng Zhu^2^, Tim Richter-Heitmann^1^, David A. Aromokeye^1,2^, Yang Liu^6,7^, Rolf Nimzyk^1^, Qingfei Zheng^5,8^, Xiaoyu Tang^5^, Marcus Elvert^2,9^, Meng Li^6,7^, Michael W. Friedrich^1,2^

^1^Microbial Ecophysiology Group, Faculty of Biology/Chemistry, University of Bremen, Bremen, Germany.

^2^MARUM - Center for Marine Environmental Sciences, University of Bremen, Bremen, Germany.

^3^Max Planck Institute for Marine Microbiology, Bremen, Germany

^4^School of Resources and Environmental Engineering, Anhui University, Hefei, Anhui, China.

^5^Institute of Chemical Biology, Shenzhen Bay Laboratory, Shenzhen, China.

^6^Archaeal Biology Center, Institute for Advanced Study, Shenzhen University, Shenzhen, China.

^7^Shenzhen Key Laboratory of Marine Microbiome Engineering, Institute for Advanced Study, Shenzhen University, Shenzhen, China.

^8^School of Chemical Biology and Biotechnology, Peking University Shenzhen Graduate School, Shenzhen, China.

^9^Faculty of Geosciences, University of Bremen, Bremen, Germany.

^*^Correspondence: Xiuran Yin, Faculty 02 (Chemistry/Biology), University of Bremen, Leobener Straße 3, D-28359, Bremen, Germany; E-mail: yin@uni-bremen.de; Tel: +49-421-218-63067.

Guowei Zhou, School of Resources and Environmental Engineering, Anhui University, 230601, Hefei, People’s Republic of China. E-mail: gwzhou@ahu.edu.cn; Tel: +86-551- 63861441.

^†^These authors contributed equally to this work.

## Supplemental Methods

**Phylogenetic analysis of gene encoding ATP citrate synthase**

For tree calculation of functional gene encoding ATP citrate synthase, backbone sequences were retrieved in UniProtKB (https://www.uniprot.org/) using the sequences of ATP citrate synthase of SG8-5 (*Ca.* Proteinoplasmatales) archaea as queries and filtered using cd-hit [1] with a cut-off of 90%, aligned by Mafft-LINSi (v7.455) [2] and trimmed by BMGE [3] with the setting of BLOSUM30. Note that all sequences from this study were included. Phylogenetic trees were constructed using IQ-TREE (1.6.12) [4] with the best-fit model and 1000 times ultrafast bootstrapping.

## Supplemental Discussion 1: Rename of active archaeal groups

Among Thermoplasmata, SG8-5 had similarities to the other subgroups less than 88.1%, 51% and 65% for 16S rRNA gene, average amino-acid and average nucleotide identities, respectively (Table S3, Fig. S8). According to the taxa descriptions for uncultured microorganisms [5], we propose *Ca.* Proteinoplasmatales as the new name for the order of SG8-5 based on demonstrated active protein utilization by representatives of this archaeal subgroup. Although we were unable to retrieve a MAG of the sister cluster of *Ca.* Proteinoplasmatales, i.e., uncultured Thermoplasmata subgroup I which was classified to Methanomassiliicoccales based on the Silva database, the 16S rRNA gene identity compared to *Ca.* Proteinoplasmatales was ~87% (Table S3) and thus this clade was named as *Ca.* Proteinoplasmatales-related (Fig. 3).

For Asgard archaea, we initially identified several OTUs that affiliated to Odinarchaeota in incubations amended with ^13^C-protein, ^13^C-DIC and streptomycin. The classification of such OTUs was supported by both Silva 132 and Silva 138 database comparisons (Fig. 3a). However, the closest 16S rRNA gene reference sequences of these Asgard archaeal OTUs had only a similarity of ~76% compared to thermophilic Odinarchaeota LCB_4 [6] (Table S3). Such low 16S rRNA gene identity was also supported by the phylogenetic tree showed in Fig. 3a, in which the OTUs and their closest references that belong to Odinarchaeota formed a sister cluster of Loki-2b archaea (Fig. 3a and Fig. S9). These tentative Odinarchaeota sequences were in fact affiliated to Lokiarchaeota as the similarity to Loki2b archaea was ~92% (Table S3). Based on these assessments, we renamed these Odinarchaeota OTUs as Loki-2c (Fig. S9).

**Supplemental Discussion 2: Potential pathway of inorganic carbon assimilation by *Ca*. Proteinoplasmatales**

Based on RNA-SIP, *Ca.* Proteinoplasmatales archaea strongly incorporated both inorganic carbon and protein-derived carbon simultaneously during protein breakdown (Fig. 2). The inorganic carbon assimilation by *Ca.* Proteinoplasmatales might have occurred during amino acid decomposition by associating with the reverse citric acid cycle (TCA) rather than the Wood-Ljungdahl pathway, which is absent in *Ca.* Proteinoplasmatales (Fig. S11). *Ca.* Proteinoplasmatales have an incomplete rTCA cycle but possesses ATP citrate synthase, the marker enzyme of rTCA [7] (Fig. 4, Fig. S12). In detail, 2-oxoglutarate, the important intermediate during amino acid degradation, might be converted to acetyl-CoA by incorporating inorganic carbon via partial rTCA.

**Supplemental Discussion 3: Living strategy of archaeal protein degraders in environments**

In long-term incubations, *Ca.* Proteinoplasmatales were detected at high abundance in the DNA-SIP samples amended with ^13^C-DIC/sulfur/lepidocrocite after 386 days (Fig. S2). Since *Ca.* Proteinoplasmatales were identified as protein degraders (Fig. 2) and they do not use sulfur as electron donor as concluded from the lack of genes involving in sulfur utilization according to metagenomic analysis [8, 9], these archaea could survive on protein of dead biomass. Similarly, since Bathy-15 do not harbor the pathways for cellulose degradation and glucose utilization (Fig. S11), their activity in the long-term SIP incubations amended with cellulose supported their living strategy on dead biomass of other microorganisms (Fig. S3).

## Supplemental Figures


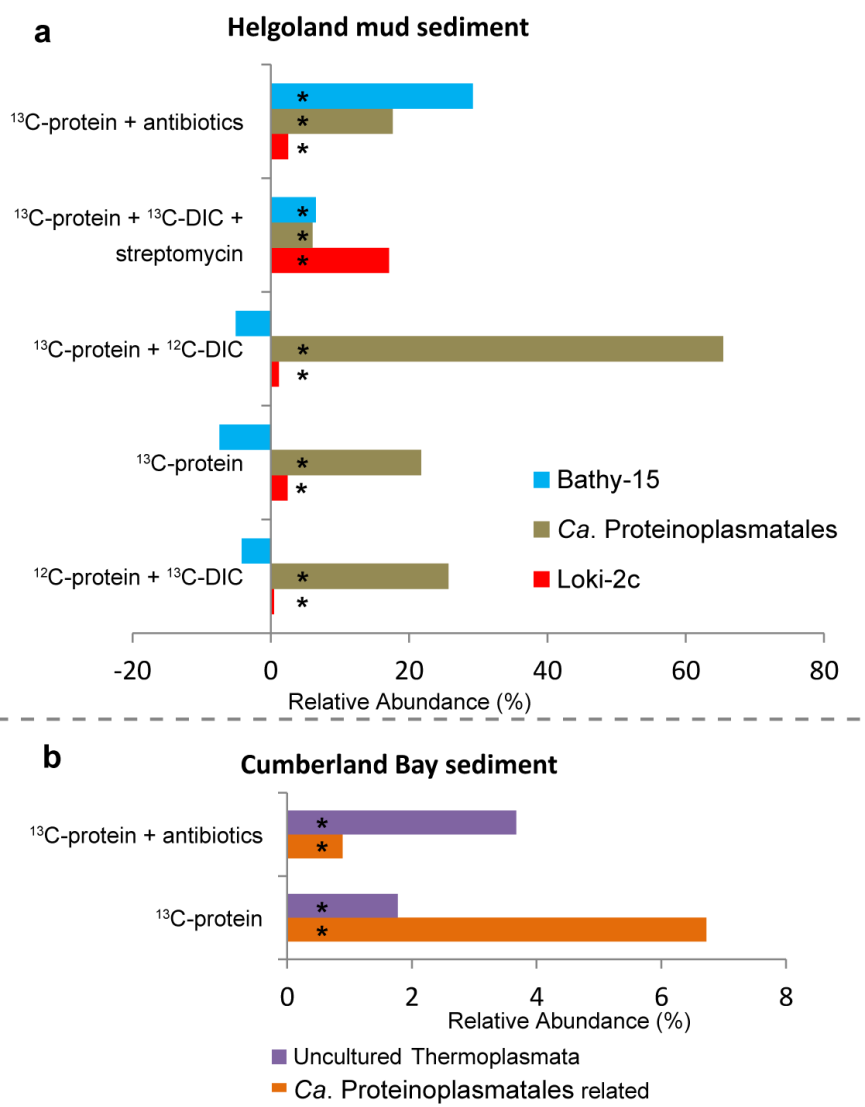


**Fig. S1** Intergradient subtraction values of relative abundance between the heavy and light fractions of RNA-SIP samples from Helgoland mud (**a**) and Cumberland Bay (**b**) sediment incubations. * indicates ^13^C-labelling of RNA in incubations amended with ^13^C-substrates.


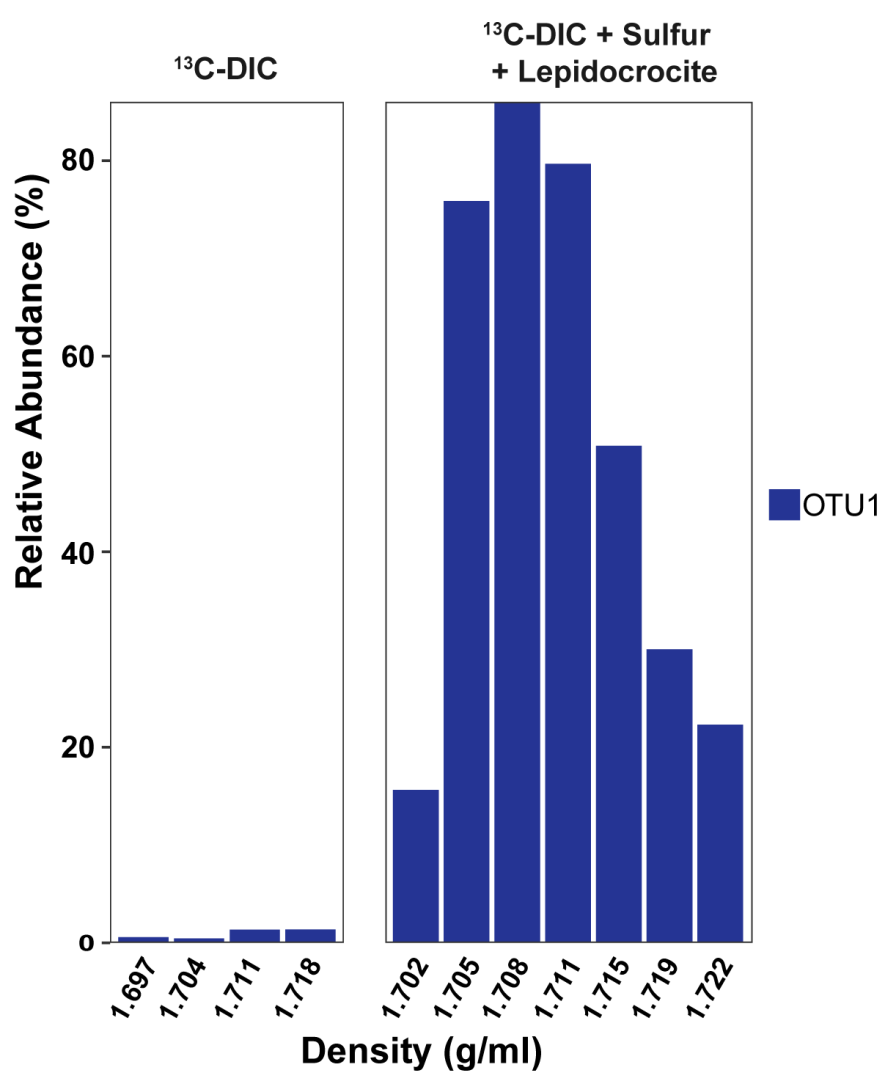


**Fig. S2** Identification of *Ca*. Proteinoplasmatales (SG8-5) by DNA-SIP in incubations amended with sulfur and lepidocrocite after 386 days (See Supplemental Discussion 3). Note that DNA has a lower density than RNA.


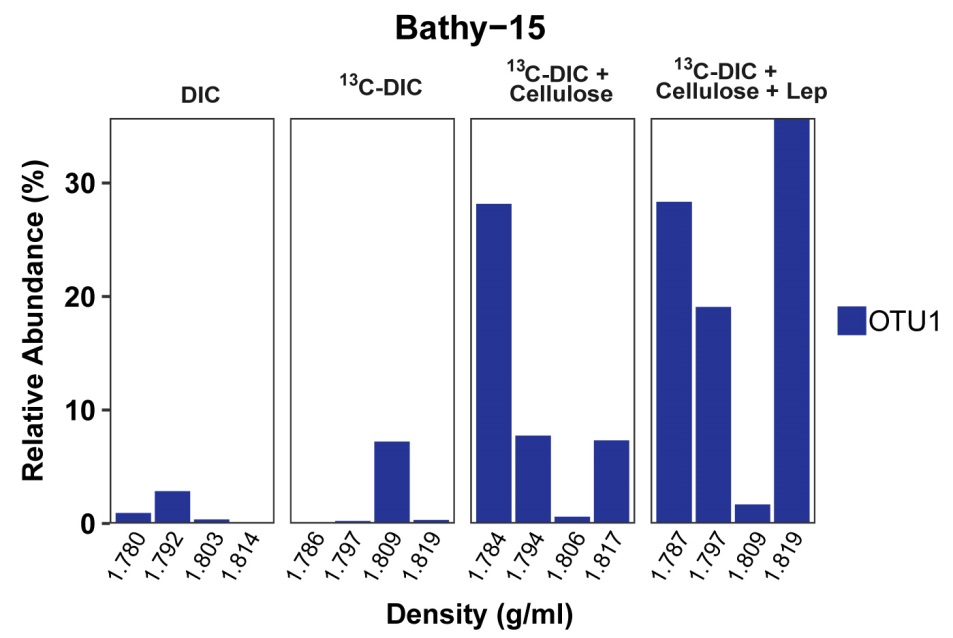


**Fig. S3** Identification of Bathy-15 by RNA-SIP in incubations amended with cellulose after 255 days. Lep: lepidocrocite. Bathy-15 were stimulated by amending cellulose and ^13^C-labelled DIC. Because high abundances of their 16S rRNA were observed in both heavy and light fractions, it is unlikely that Bathy-15 used inorganic carbon as carbon source (See Supplemental Discussion 3).

**
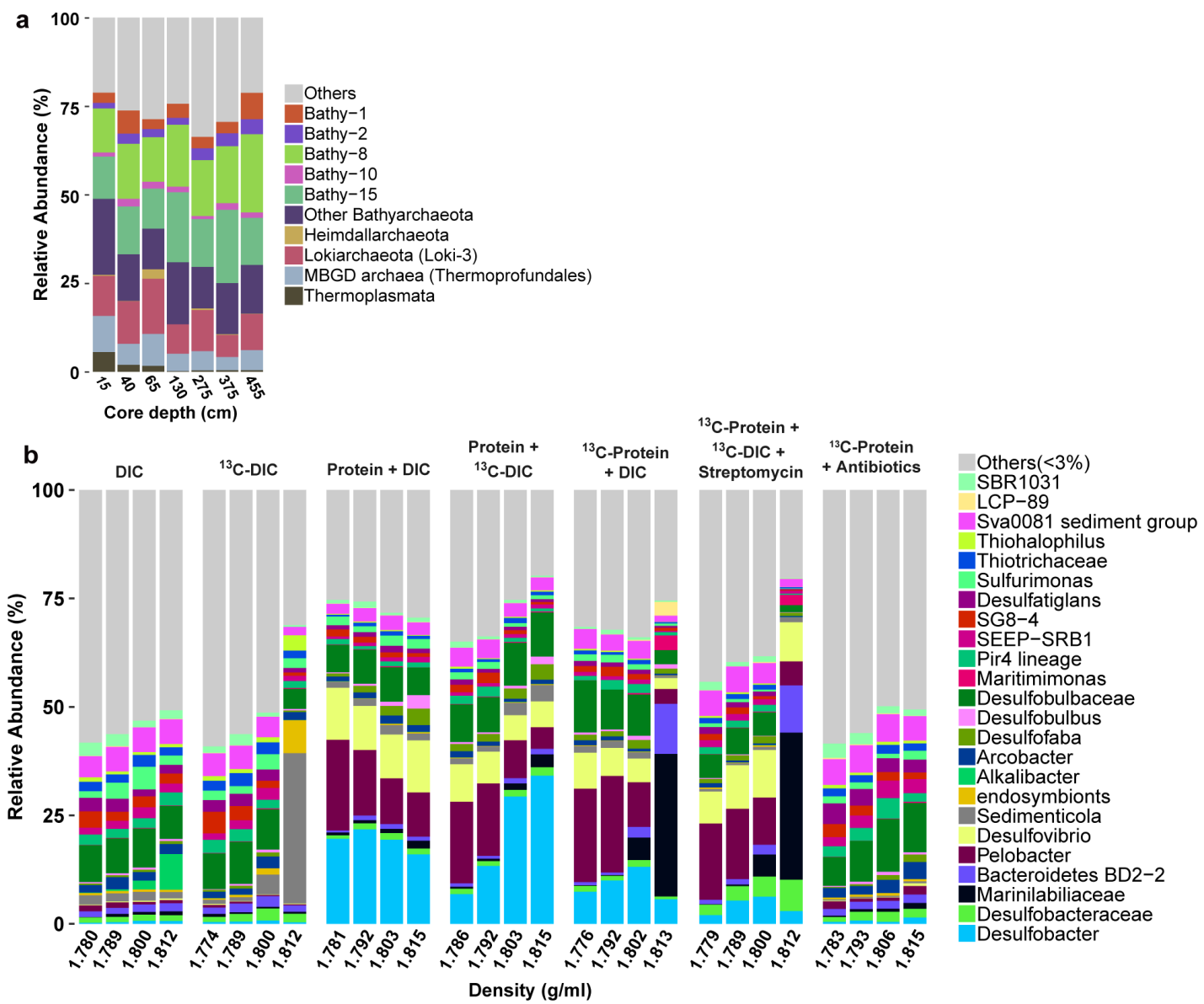
**

**Fig. S4**  Relative abundance of 16S rRNA gene sequences of archaea in original sediment **(a)** and **(b)** bacteria from RNA-SIP gradient fractions in the Helgoland mud sediment incubations.


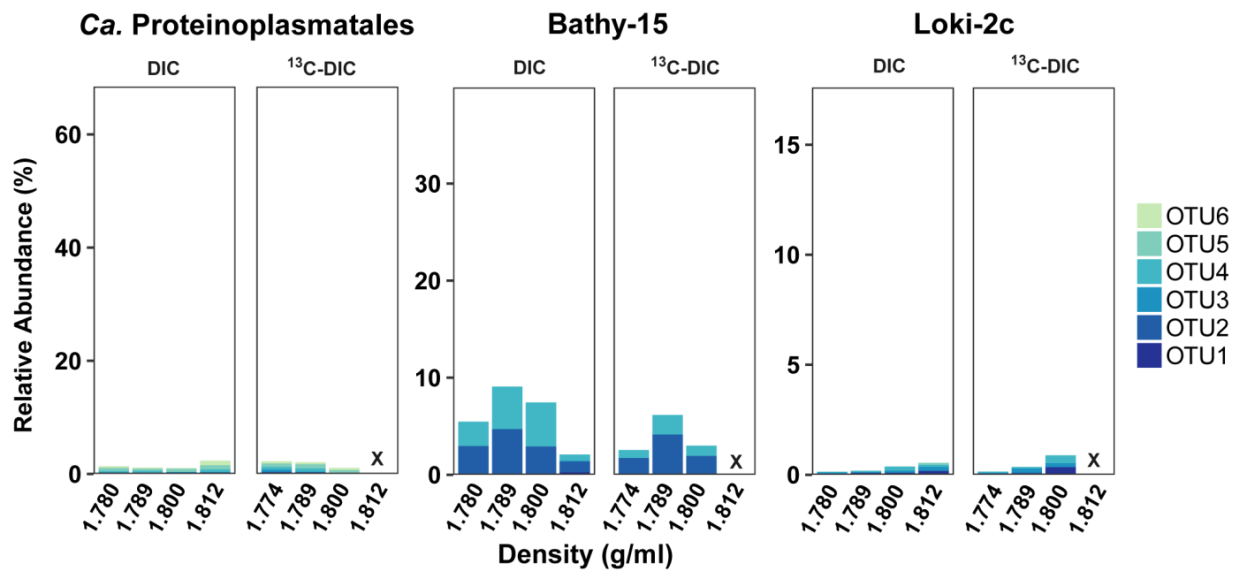


**Fig. S5** Unlabelled and ^13^C-labelled DIC control incubations for RNA-SIP samples using Helgoland mud sediment. X indicates below detection due to the low number of reads obtained from 16S rRNA gene sequencing.


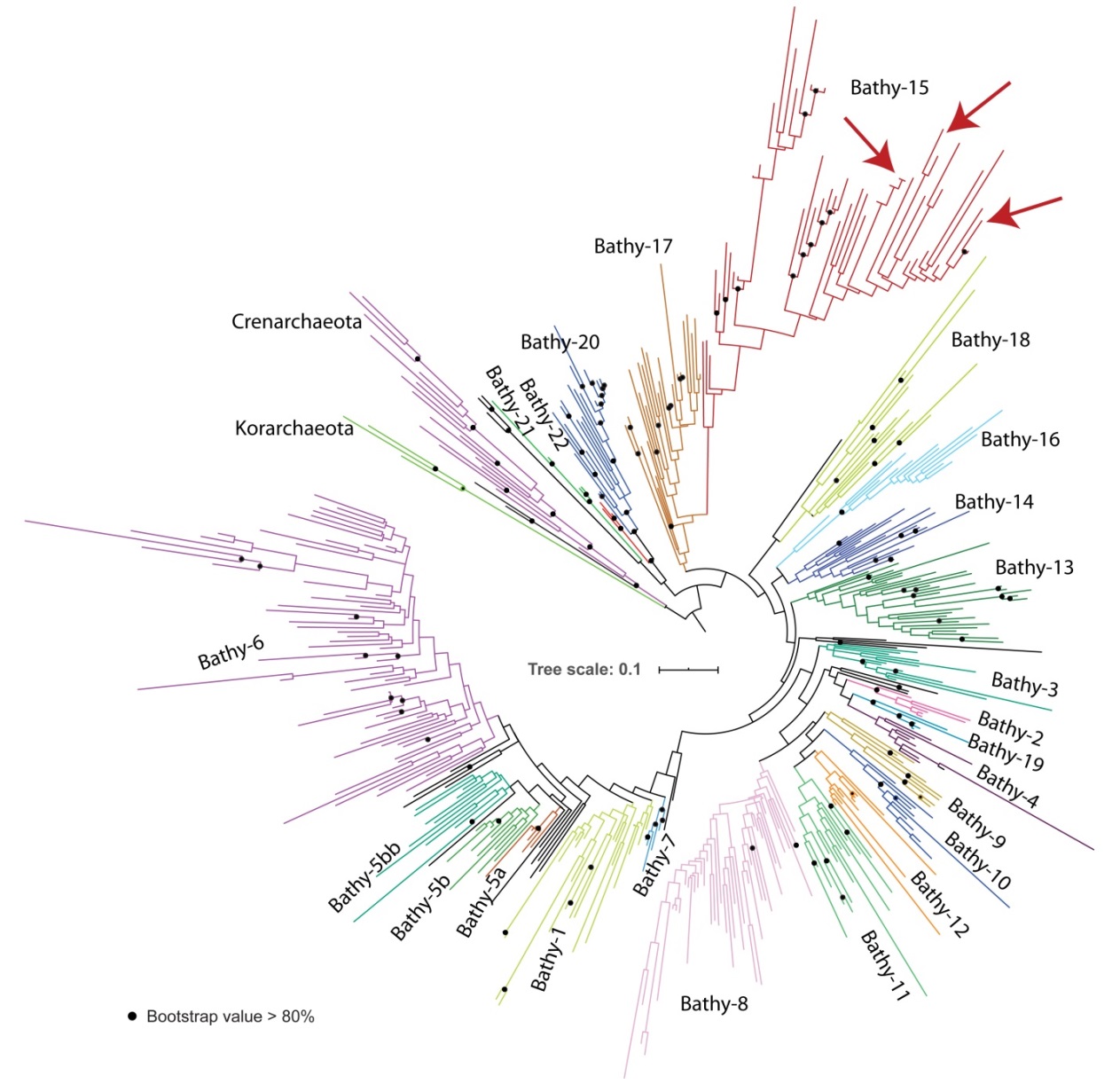


**Fig. S6** RAxML tree of Bathy-15 including sequences detected in incubations with Helgoland mud sediments. Arrow indicates the 16S rRNA gene OTUs detected in this study.


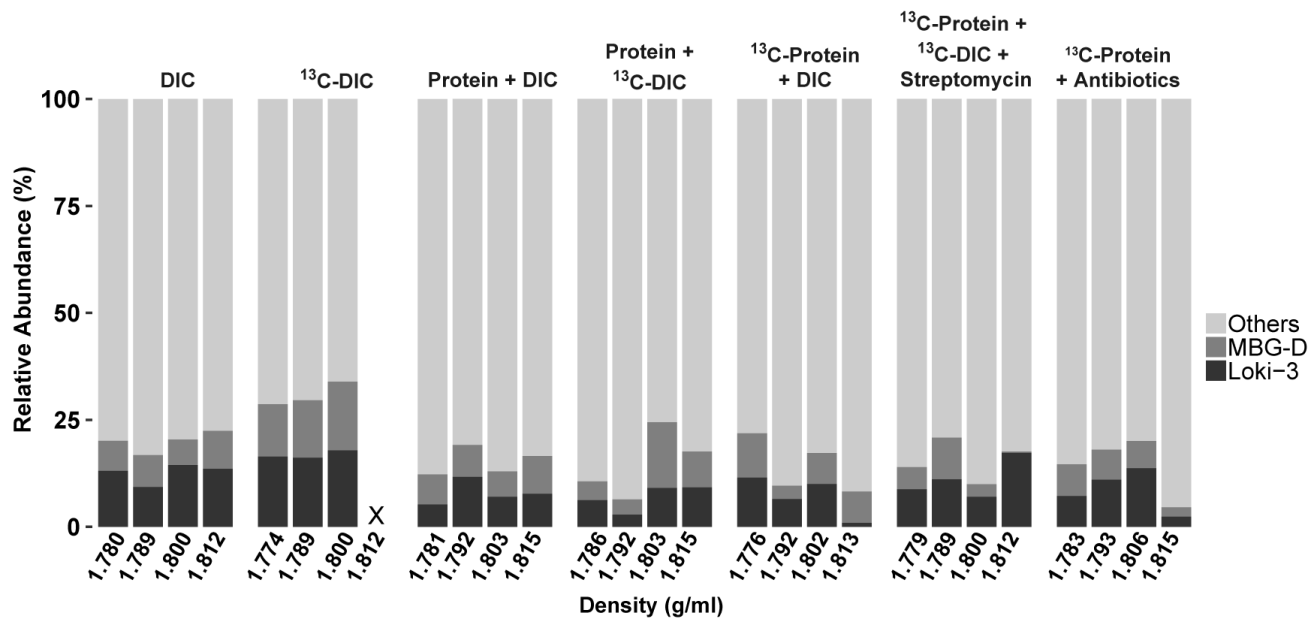


**Fig. S7** Relative abundance of Loki-3 in SIP samples amended with protein. X indicates below detection due to the low number of reads obtained from 16S rRNA gene sequencing.


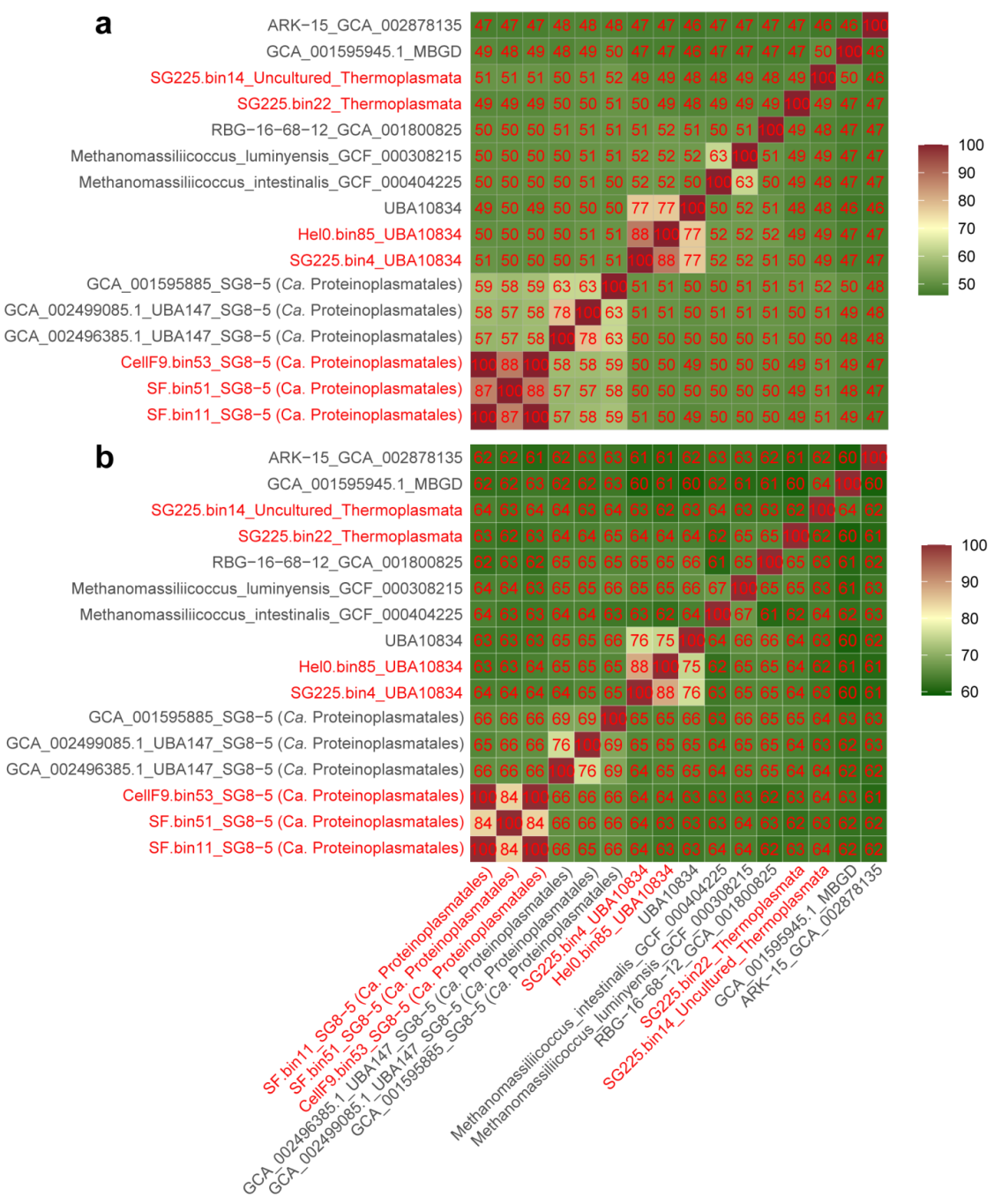

**Fig. S8** Comparison of average amino acid identity (AAI) (**a**) and average nucleotide identity (ANI) (**b**) between *Ca*. Proteinoplasmatales (SG8-5) and other Thermoplasmata. Archaeal names marked in red indicate MAGs retrieved in this study.


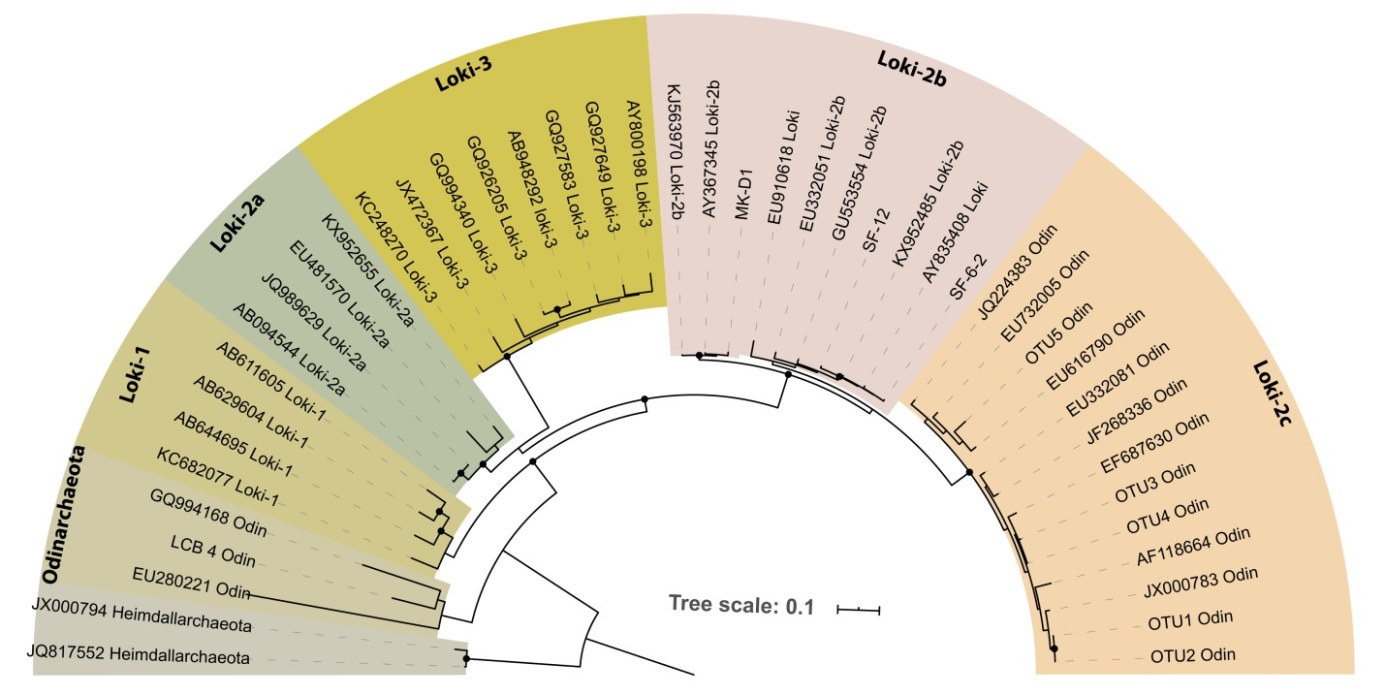
**Fig. S9** Maximum likelihood tree of archaeal 16S rRNA genes within Asgard archaeal groups.SF-12 and SF-6-2 are Loki-2b clones retrieved previously[9].

**
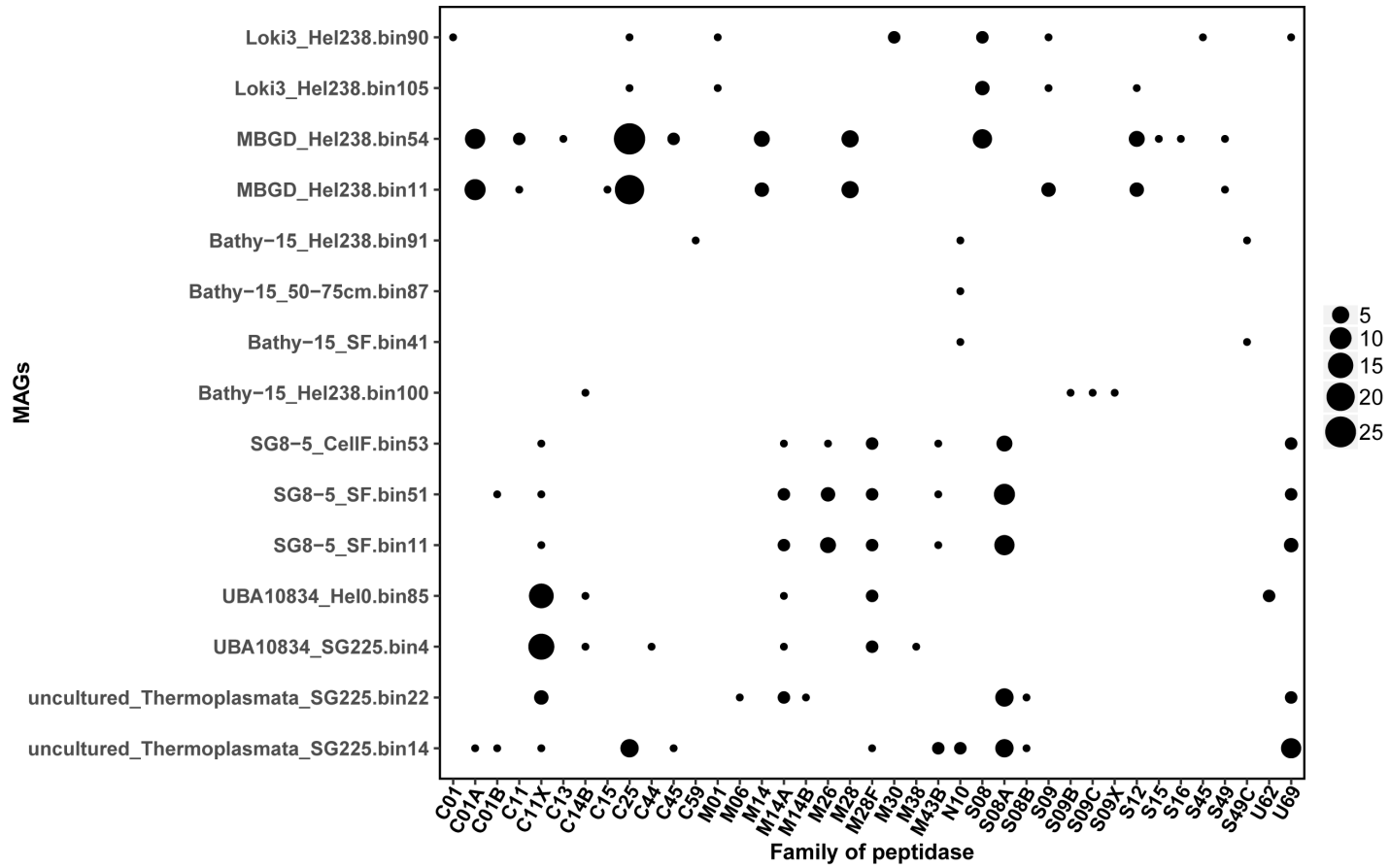

Fig. S10** Distribution of peptidase families for genes encoding extracellular peptidases identified in archaeal MAGs in this study. Number of genes indicated by size of filled circles.


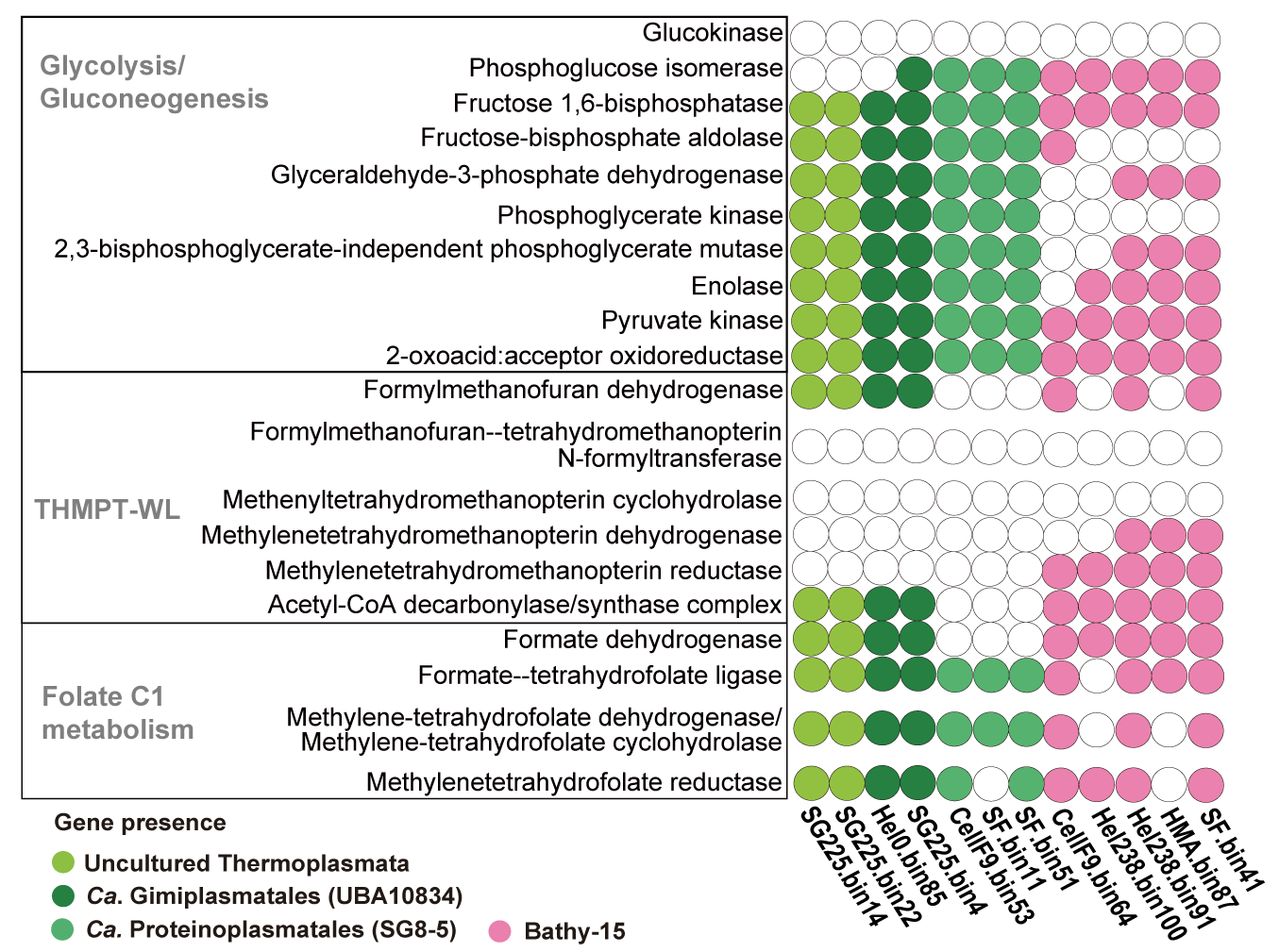


**Fig. S11** Presence of genes coding for glycolysis, Wood–Ljungdahl pathway and folate C1 metabolism in MAGs of protein degrading archaea.


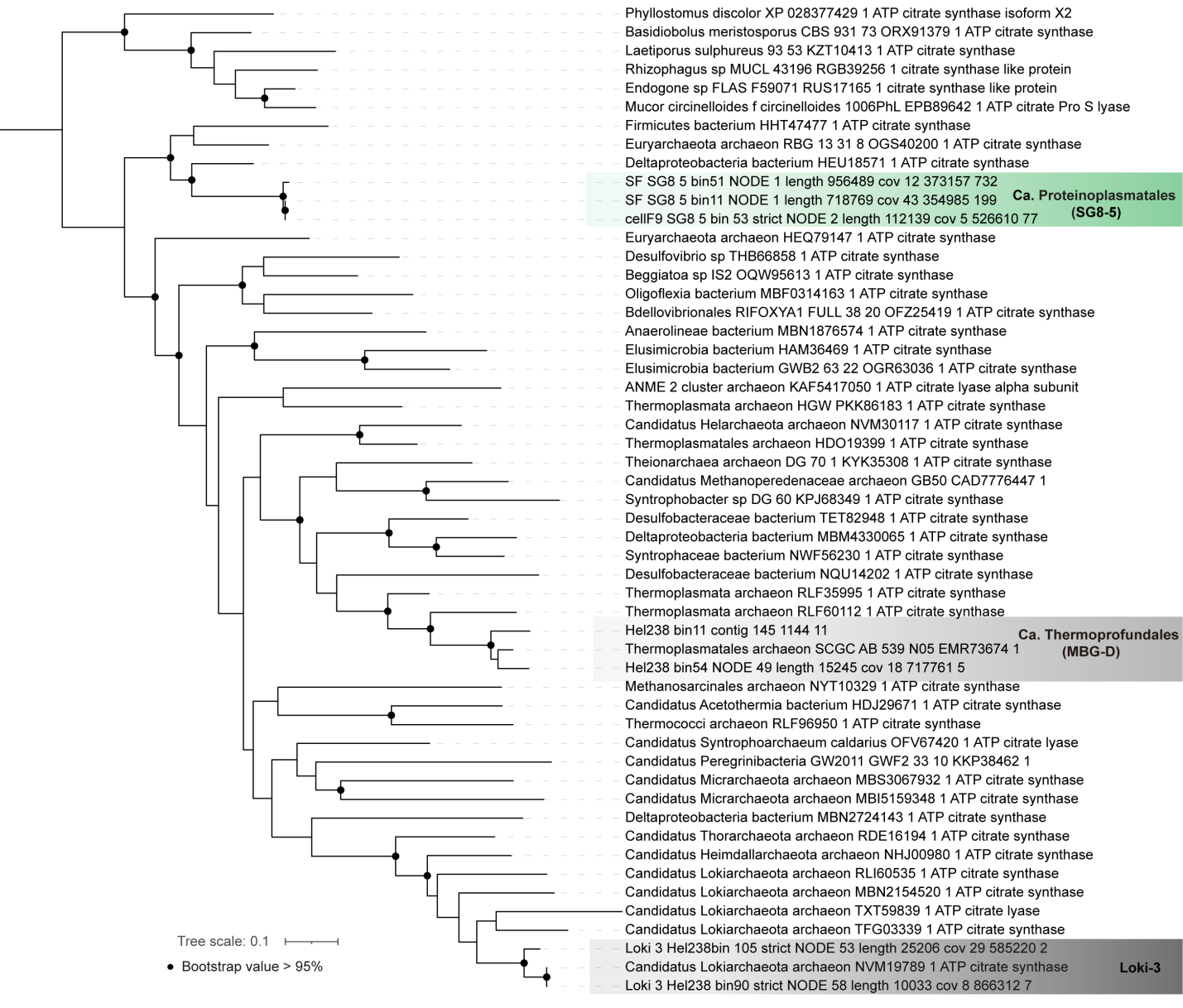


**Fig. S12** Maximum likelihood phylogenetic analyses of ATP citrate synthase in *Ca.* Proteinoplasmatales (SG8-5), MBG-D and Loki-3 archaea. The phylogeny inferred from an alignment consisting of 608 amino acid positions with the best-fit model LG+I+G4.


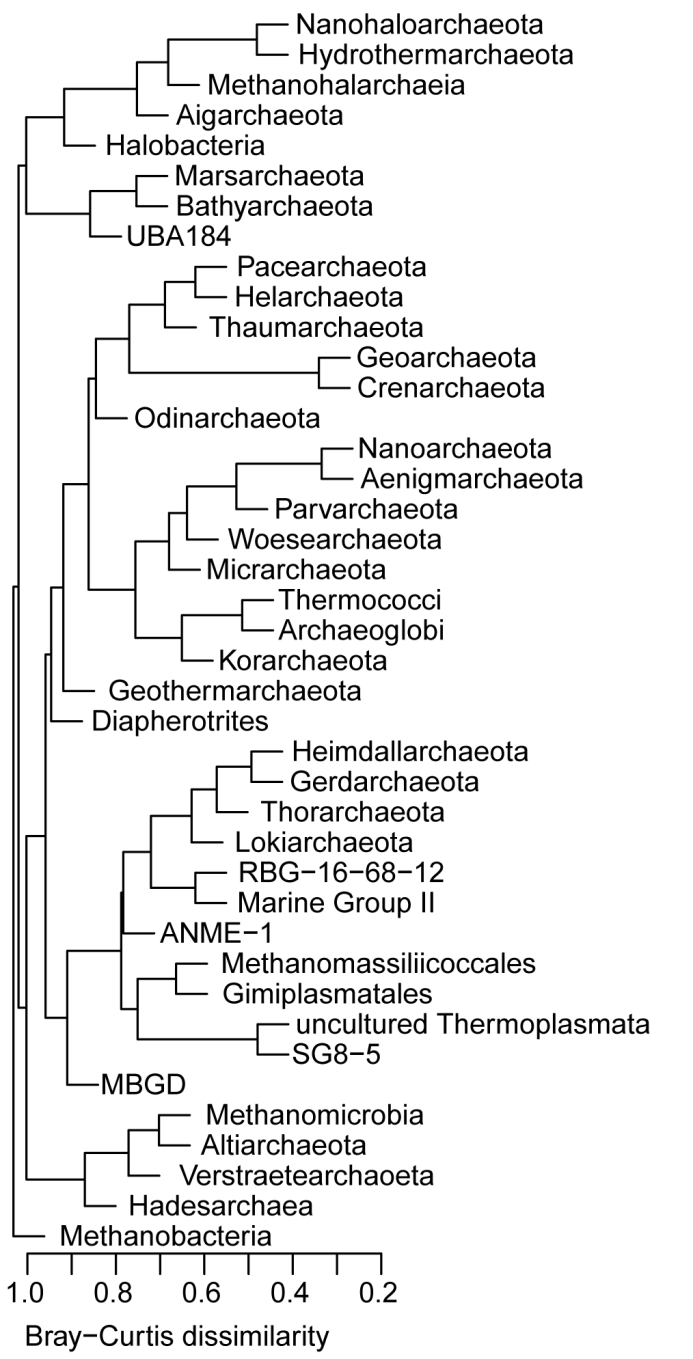


**Fig. S13** Bray-Curtis dissimilarity for extracellular peptidase family among divergent archaeal groups based on 180 MAGs. Bray-Curtis dissimilarities were calculated from the extracellular peptidase gene abundance table across all MAGs (Table S5).

**Reference**

1. Fu L, Niu B, Zhu Z, Wu S, Li W. CD-HIT: accelerated for clustering the next-generation sequencing data. Bioinformatics. 2012;28:3150-2.

2. Katoh K, Standley DM. MAFFT multiple sequence alignment software version 7: improvements in performance and usability. Mol Biol Evol. 2013;30:772-80.

3. Criscuolo A, Gribaldo S. BMGE (Block Mapping and Gathering with Entropy): a new software for selection of phylogenetic informative regions from multiple sequence alignments. BMC Evol Biol. 2010;10:210.

4. Nguyen LT, Schmidt HA*,* von Haeseler A, Minh BQ. IQ-TREE: a fast and effective stochastic algorithm for estimating maximum-likelihood phylogenies. Mol Biol Evol. 2015;32:268-74.

5. Konstantinidis KT, Rossello-Mora R, Amann R. Uncultivated microbes in need of their own taxonomy. ISME J. 2017;11:2399-406.

6. Spang A, Stairs CW, Dombrowski N, Eme L, Lombard J, Caceres EF, et al. Proposal of the reverse flow model for the origin of the eukaryotic cell based on comparative analyses of Asgard archaeal metabolism. Nat Microbiol. 2019;4:1138–48.

7. Hugler M, Huber H, Molyneaux SJ, Vetriani C, Sievert SM. Autotrophic CO_2_ fixation via the reductive tricarboxylic acid cycle in different lineages within the phylum Aquificae: evidence for two ways of citrate cleavage. Environ Microbiol. 2007;9:81-92.

8. Imachi H, Nobu MK, Nakahara N, Morono Y, Ogawara M, Takaki Y, et al. Isolation of an archaeon at the prokaryote-eukaryote interface. Nature. 2020;577:519-25.

9. Yin X, Cai M, Liu Y, Zhou G, Richter-Heitmann T, Aromokeye DA, et al. Subgroup level differences of physiological activities in marine Lokiarchaeota. ISME J. 2020;15:848–61.
